# Public awareness, knowledge, and attitude toward epilepsy in Lebanon: a cross-sectional study

**DOI:** 10.3389/fneur.2024.1480960

**Published:** 2024-12-11

**Authors:** Samar Younes, Bahia Chahine, Venise Hanna, Nisreen Mourad, Farah Al Souheil, Nadia Al Mazrouei, Mohamad Rahal, Diana Malaeb

**Affiliations:** ^1^School of Pharmacy, Lebanese International University, Beirut, Lebanon; ^2^INSPECT-LB (Institut National de Santé Publique, d’Épidémiologie Clinique et de Toxicologie-Liban), Beirut, Lebanon; ^3^Inserm U1094, IRD UMR270, University of Limoges, EpiMaCT Epidemiology of Chronic Diseases in Tropical Zone, Limoges, France; ^4^IVPN-Network, Fujairah, United Arab Emirates; ^5^Department of Pharmacy Practice and Pharmacotherapeutics, College of Pharmacy, University of Sharjah, Sharjah, United Arab Emirates; ^6^College of Pharmacy, Gulf Medical University, Ajman, United Arab Emirates

**Keywords:** epilepsy, awareness, knowledge, attitudes, public, Lebanon

## Abstract

**Purpose:**

Epilepsy, a prevalent chronic neurological disorder, is associated with misconceptions, negative attitudes, and stigma because of poor public knowledge and awareness. Therefore, the purpose of the current study was to assess the knowledge, awareness, and attitudes toward epilepsy and its associated factors among the Lebanese general population.

**Methods:**

A cross-sectional study was conducted on Lebanese adults between October 2021 and June 2022 using an electronic structured self-administered questionnaire that was circulated online using the different social media platforms. Data related to participants’ demographic characteristics, knowledge, awareness, and attitude about epilepsy were measured. Eight items were included in the knowledge score that ranged between “0 and 16”, five items were included in the awareness score and ranged between 0 and 10. Likewise, the attitude score was calculated from 14 items and the score ranged between “0 and 28”. Data was analyzed using descriptive statistics, and logistic regression was employed to identify factors associated with knowledge and awareness of epilepsy.

**Results:**

A total of 301 participants filled the questionnaire with a mean age of 28.68 and majority (66%) were females. The analyzed scores revealed that most of the participants (87.4%) had a good knowledge regarding epilepsy as a disease with mean of 10.2 (± 2.14), 70.1% had good awareness about epilepsy with mean of 6.3 (± 1.4), and 88% had good attitude toward patients with epilepsy with mean of 21.5 (± 6.2). Attending lecture about epilepsy was significantly associated with higher knowledge (OR 5.756, CI 95% 4.652–11.676, *p* < 0.001), better awareness (OR 4.936, CI 95% 3.062–10.566, *p* < 0.001) and attitude (OR 5.187, CI 95% 1.687–9.761, *p* < 0.014) toward the disease.

**Conclusion:**

The findings of this study reflected an adequate knowledge and awareness, as well a positive attitude toward patients with epilepsy in Lebanon. However, there is a need for raising societal awareness and understanding of epilepsy to minimize the public misconceptions and reduce the stigma and discriminatory attitudes toward epileptic patients.

## Introduction

Epilepsy, a prevalent stigmatizing neurological illness, is characterized by recurrent unprovoked seizures resulting from abnormally hypersynchronous and hyper-excited neuronal activity in the brain (1, 2). It affects individuals irrespective of their ages, nationalities, ethnicities, socioeconomic groups, and geographic locations (3), thus leading to the emergence of transient and various signs and symptoms (4). Epilepsy, considered a global disease, is associated with neurological, cognitive, psychological, and social implications (5). According to the WHO, at least 50 million individuals worldwide suffer from epilepsy in 2019, with almost 80% living in low- and middle-income countries (6, 7). It is worth mentioning that epileptic patients have a threefold increased chance of dying prematurely compared to the general population (8). Furthermore, a systematic review of the epidemiology of epilepsy in Arab countries in 2009 showed that around 724,500 people have epilepsy (9); nevertheless, there is a paucity of data on the epidemiology of the disease in Lebanon.

Epilepsy has long been recognized as a disorder that requires public health intervention; however, the focus in the past was primarily on one aspect of epilepsy management, that is episodes of seizure control. In this regard, minimal efforts were concerned with the disorder’s social, contextual, and cultural consequences, as well as its knowledge which is often based on the public perceptions (10). Notably, epileptic patients have substantial negative social and psychological effects (e.g., anxiety, embarrassment, and limitations in social relationships) that result from the wrong perceptions and beliefs of the disorder (11, 12). These misconceptions and negative public attitudes toward epilepsy may possibly arise from the absence of solid factual information about epilepsy (10, 13), subsequently leading to a stigma that is widespread across many cultures (14). Epilepsy is triggered by different key risk factors including but not limited to: positive family history, prior head traumas and tumors, previous stroke or vascular diseases, dementia, fever, and infections (15). As a consequence, epileptic patients often confront difficulties in leading an active life and surviving its individual phases: education, employment and marriage (16). Dealing with this stigma and common public misunderstandings is commonly regarded as one of the greatest obstacles which tend to negatively impact the lives of epileptic patients and their families. Thus, the discrimination and stigma ultimately deter the patients from seeking appropriate care, leading to delayed diagnosis and inappropriate treatment (2, 17, 18).

Knowledge about epilepsy involves understanding of various key elements that are known to influence disease progression and complications. Thus, good knowledge mandates awareness about the different types of epilepsy, triggers, warning signs, treatment strategies, medication options, and emergency plan (19). In Lebanon, cross-sectional studies were conducted among epileptic patients and revealed that good knowledge of epilepsy and social support were important predictors of good quality of life (20, 21). However, measuring awareness among the public is also vital for obtaining an overall view and perception of epilepsy and improving this condition (13). Additionally, public knowledge about epilepsy is another significant component in minimizing the impact of seizures, improving the health-related quality of life for the patients and their families, as well as reducing the stigma and discriminatory attitudes toward epileptic patients (11, 22). According to previous data from the literature, raising knowledge about epilepsy level is associated with low level of epilepsy-related stigma (23–25). Hence, understanding the degree of community knowledge, attitudes, and perceptions exerts a substantial impact on the educational programs and awareness campaigns which in turn improve community understanding of epilepsy (26). Furthermore, despite the decrease in epilepsy burden from 1990 to 2016 in different countries including Lebanon, yet it is still an important cause of disability and mortality (3). Policymakers, the district health team, community members, affected families, and epileptic patients could also indirectly benefit from the application of appropriate strategies (27). Therefore, the current study was conducted to assess the knowledge, awareness, and attitudes toward epilepsy and its associated factors among the Lebanese general population.

## Methods

### Study design and population

This is a cross-sectional study that was conducted between October 2021 and June 2022 using a questionnaire that was designed based on questions adapted from two studies (28, 29). The questionnaire was created using Google Forms, a cloud-based survey tool powered by Google™ and its link was circulated online using the different social media platforms (e.g., WhatsApp, Facebook, and Instagram), using snowball sampling to enroll participants. Additionally, participants were encouraged to share the survey link within their personal networks to facilitate broader outreach. Consent to participate was obtained before completing the online survey in accordance with the Lebanese International University- Institutional Review Board regulations which provided the ethical approval of this study (2021RC-042-LIUSOP).

Only Lebanese adults (i.e., aged between 18 and 65 years) were included in this study. Healthcare personnel and students enrolled in any of the healthcare majors (medicine, pharmacy, nursing, midwifery, pharmacy, and lab technicians) were excluded.

### Sample size calculation

Data from Muthaffar and Jan (30) study in Saudi Arabia revealed that around 77.4% of observed participants had prior knowledge of epilepsy. Using the Epi Info software, a minimum sample of 269 respondents was estimated sufficient to be included in this study for robust results with a significance level of 5%.

### Data collection

The questionnaire used in this study was adapted from validated tools assessing knowledge, awareness, and attitudes toward epilepsy (28, 29). It was first translated into the local language (Arabic) following a standard forward and backward translation process to ensure accuracy and cultural relevance. Both the English and Arabic versions were reviewed by experts in the field. The participants were given the option to answer the questionnaire in either English or Arabic, depending on their preference, to ensure better comprehension and more accurate responses. The study questionnaire included questions about the participants’ demographics (i.e., age, gender, place of residence, marital status, education, occupation, income), medical problems (having ever been diagnosed by a healthcare provider with a long-term condition or currently taking medication for such a condition, with examples including hypertension, diabetes, cardiovascular diseases, asthma, and chronic obstructive pulmonary disease), social history (alcohol consumption and smoking status referred to *current use*; alcohol consumption was defined as participants who reported drinking alcohol in the past month, and smoking status referred to those who were actively smoking at the time of the survey), and insurance type (national social security fund (NSSF), cooperation healthcare fund (COOP), private, no insurance). This first part also included a question exploring if the participant previously attended any lecture or seminar about epilepsy as well as a question to assess participants’ self-perceived level of knowledge about epilepsy. The remaining sections of the questionnaire included questions that assessed the participants’ knowledge, awareness, perception, and attitude toward epilepsy, as well as a question to assess participants’ feeling toward an individual having a seizure. The average completion time for the questionnaire was approximately 7–10 min.

Eight items were included in the knowledge score where four of them included multiple responses: a “yes” response was coded by “2” and “no” was coded by “1” and “I do not know” were coded by “0.” Thus, the score ranged between “0 and 16” with “0 and 8” indicating “poor knowledge” while a score between “9 and 16” indicating “good knowledge.” The Cronbach’s alpha for the knowledge score was 0.65. These items included questions about epilepsy etiology, clinical manifestations, treatment options, and how to help patients having epilepsy. It also assessed participants’ knowledge whether epilepsy is contagious, lifelong, curable, or dangerous disease, Similarly, five items were included in the awareness score; the score ranged between 0 and 10: “0–5” indicating “poor awareness” and “6–10” representing “good awareness.” The Cronbach’s alpha for the awareness score was 0.61. Items included: witnessing epilepsy, having a relative with epilepsy, having a close friend with epilepsy, having a work or classmate with epilepsy, as well as awareness of the existing types of epilepsy. Likewise, the attitude score was calculated from 14 items and the score ranged between “0 and 28”, where a score of “0–16” indicated a “negative attitude” and a score of “17–28” indicated a positive one. Our study defined a “good attitude” toward patients with epilepsy based on specific positive responses to attitudinal questions included in the questionnaire. These questions were designed to gauge participants’ perceptions and willingness to support individuals with epilepsy. For instance, they included: “Do you think that patients with epilepsy are intelligent like other people?,” “Do you think that patients with epilepsy are insane/mentally ill?,” “Do you shake hands with a patient with epilepsy?,” “Do you allow your son/daughter to marry a girl with epilepsy?,” “Do you think that patients with epilepsy can have children?,” etc. The Cronbach’s alpha for the attitude score was 0.87.

### Statistical analysis

Data were analyzed using the Statistical Package for the Social Sciences (Version 25). Descriptive statistics were used for the participants’ demographics and other characteristics; mean and standard deviation described continuous variables, while frequencies and percentages were used to analyze categorical variables. The Chi-square test was used to determine the association between the scores of knowledge, awareness, and attitude and categorical variables. Variables that showed a *p* < 0.2 in the bivariate analysis were entered as independent variables in the regression models. Statistical significance was set at a *p* ≤ 0.05. In the logistic regression analysis, education was dichotomized into “postgraduate” or “Bachelor.” Occupation was dichotomized into “employed” vs. “unemployed” (retired, student, unemployed). Insurance was dichotomized into “insured” (NSSF, COOP, private) vs. “noninsured.” Residency was dichotomized into “Bekaa” vs. “others” (all remaining regions), based on the number of participants.

## Results

**Figure 1:**
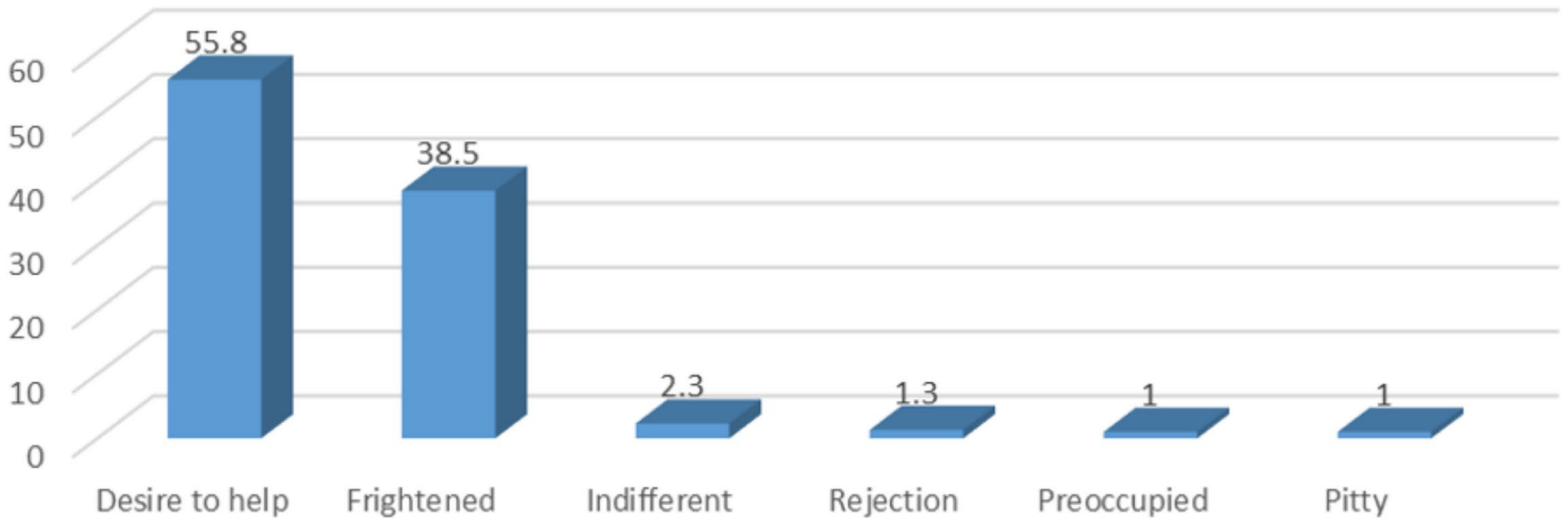
Feeling towards an individual having a seizure.

A total of 478 participants completed the questionnaire of which 301 were eligible and included in the study. The participants’ sociodemographic characteristics are displayed in Table 1. The mean age of the participants was 28.68 years (± 9.6) and 199 (66.1%) were females. Half of the participants earned a university degree 154 (51.2%) and 139 (46.2%) were unemployed. Only 72 (23.9%) of the participants attended lectures about epilepsy, and 158 (52.5%) believed that the information they currently have on epilepsy is insufficient. Participants’ knowledge about epilepsy is demonstrated in Table 2. The major etiologies of epilepsy were believed to be genetic 317 (66.3%), brain disease 180 (59.8%), and toxic substance or medication 169 (56.1%). Regarding the clinical presentations of epilepsy, convulsion/shaking 227 (75.4%), sudden loss of consciousness 202 (67.1%), and behavioral change 161 (53.5%) were the most known. Most of the participants reported that epilepsy is not contagious 254 (84.4%). Table 3 shows the self-reported awareness and experience of the participants regarding epilepsy. Less than half 145 (48.2%) stated that they witnessed epilepsy. However, only a quarter 78 (25.9%) were aware of the types of epilepsy present. The participants’ attitudes toward epilepsy are presented in Table 4. More than half 204 (67.8%) assumed that patients with epilepsy are mentally ill, and 199 (33.2%) reported that these patients have a greater risk of developing mental disorders. Less than half 145 (48.3%) agreed that epileptic patients should not be allowed to drive, whereas 226 (75.1%) assumed that they could participate in sport activities. Participants’ feeling toward an individual having a seizure are presented in Figure 1. When calculating the knowledge, awareness, and attitude scales’ scores, most of the participants 263 (87.4%) had a good knowledge regarding epilepsy as a disease with mean score of 10.2 ± 2.14, 211 participants (70.1%) had good awareness with mean score of 6.3 ± 1.4, and 263 participants (88%) had good attitude toward patients with epilepsy with mean score of 21.5 ± 6.2. Factors associated with the knowledge, awareness, and attitude scores are presented in Table 5. The results of the bivariate analysis showed that a variation in participants’ knowledge scores was significantly associated with the employment status of the individual (*p* = 0.015). Moreover, attending a lecture about epilepsy was significantly associated with the participants’ level of knowledge (*p* < 0.001), awareness (*p* < 0.001), and attitude (*p* = 0.006). Furthermore, participants’ level of awareness was significantly associated with their place of residence (*p* = 0.011). Participants’ attitude toward epilepsy was also significantly associated with their educational level (*p* = 0.023) and occupation (*p* = 0.027), where those having a positive attitude toward the disease were more likely to have a university degree. Table 6 shows the logistic regression analysis. Attending lecture about epilepsy was significantly associated with higher knowledge (OR 5.756, CI 95% 4.652–11.676, *p* < 0.001), better awareness (OR 4.936, CI 95% 3.062–10.566, *p* < 0.001) and attitude (OR 5.187, CI 95% 1.687–9.761, *p* < 0.014) toward the disease. Moreover, higher age (OR 4.493, CI 95% 1.182–7.075, *p* < 0.027) was significantly associated with better attitude.

**Table 1:** Participants’ characteristics; *n* (%) unless otherwise stated (*N* = 301).

Characteristic		*n* (%)
Age (years)	18–30	202 (67.1)
	31–40	65 (21.6)
	41–65	34 (11.3)
Age range (years), mean ± standard deviation		29 (10)
Gender	Males	102 (33.9)
	Females	199 (66.1)
Place of residence	Bekaa	151 (50.2)
	Beirut	44 (14.6)
	Mount Lebanon	31 (10.3)
	Baalbek/Hermel	22 (7.3)
	South	20 (6.6)
	Akkar	14 (4.7)
	North	13 (4.3)
	Nabatieh	6 (2)
Marital status	Single	183 (60.8)
	Married	107 (35.5)
	Divorced	11 (3.7)
Education	Primary	7 (2.3)
	Intermediate	7 (2.3)
	Secondary	38 (12.6)
	University	154 (51.2)
	Postgraduate	95 (31.6)
Occupation	Work in non-healthcare	132 (43.9)
	Unemployed	139 (46.2)
	Retired	6 (2)
	Student in non-healthcare	24 (8)
Current alcohol consumption	Yes	18 (6)
	No	283 (94)
Smoking status	Current smoker	106 (35.2)
	Non-smoker/Former smoker	195 (64.8)
Monthly household income (Lebanese pounds)	< 750,000	21 (7)
	750,000–2,000,000	110 (36.5)
	2,000,001–4,000,000	106 (35.2)
	> 4,000,000	64 (21.3)
Insurance type	NSSF*	77 (25.6)
	Private	77 (25.6)
	COOP**	10 (3.3)
	No insurance	137 (45.5)
Chronic illness	Yes	54 (17.9)
Attended a lecture about epilepsy	Yes	72 (23.9)
Self-perceived level of knowledge on epilepsy	Advanced	20 (6.6)
	Average	91 (30.2)
	Insufficient	158 (52.5)
	None	32 (10.6)

^*^NSSF, national social security fund. **COOP, cooperation healthcare fund.

**Table 2:** Knowledge of epilepsy among observed participants *n* (%) (*n* = 301).

Knowledge attribute		*n* (%)
Etiology	Brain disease	180 (59.8)
	Complications during childbirth	152 (50.5)
	Toxic substances and medications	169 (56.1)
	Genetic	198 (65.8)
	High fever	71 (23.6)
	Accident	162 (53.8)
	Stroke	104 (34.6)
Clinical manifestations	Convulsions or shaking	227 (75.4)
	Sudden loss of consciousness	202 (67.1)
	Behavioral change	161 (53.5)
	Period of memory disturbance	109 (36.2)
How to help patient having a seizure	Hold tongue	111 (36.9)
	Protect head	168 (55.8)
	Remove nearby sharp objects	182 (60.5)
	Call an ambulance	176 (58.5)
	Stay close to individual	93 (30.9)
	Restrict movement	126 (41.9)
Treatment options	Medications	272 (90.4)
	Surgery	156 (51.8)
	Special food/diet	69 (22.9)
	Traditional or herbal medications	30 (10)
Is epilepsy contagious	Yes	16 (5.3)
	No	254 (84.4)
	Do not know	31 (10.3)
Is epilepsy a lifelong disease	Yes	185 (61.5)
	No	82 (27.2)
	Do not know	34 (11.3)
Is epilepsy curable	Yes	68 (22.6)
	No	165 (54.8)
	Do not know	68 (22.6)
Is epilepsy a dangerous disease	Yes	144 (47.8)
	No	128 (42.5)
	Do not know	29 (9.6)

Knowledge score = 8 items (four questions with multiple responses), score range 0–16 (0–8 = poor knowledge, 9–16 = good knowledge).

**Table 3:** Participants’ self-reported awareness and experience with epilepsy (*n* = 301).

Variable	Yes, *n* (%)	No, *n* (%)
Witnessed epilepsy	145 (48.2)	156 (51.8)
Have a relative with epilepsy	114 (37.9)	187 (62.1)
Have a close friend with epilepsy	74 (24.6)	227 (75.4)
Have a work or class mate with epilepsy	41 (13.6)	260 (86.4)
Awareness of how many types of epilepsy exist	78 (25.9)	223 (74.1)

Awareness score (5 items, no = 1, yes = 2) score range 0–10 (0–5 = poor awareness, 6–10 = good awareness).

**Table 4:** Attitude of observed participants towards epilepsy *n* (%) (*n* = 301).

Variable	Yes	No	Do not know
1. Do you think that patients with epilepsy are intelligent like other people?	251 (83.4)	21 (7)	29 (9.6)
2. Do you think that patients with epilepsy are insane/mentally ill?	204 (67.8)	31 (10.3)	66 (21.9)
3. Do you think that patients with epilepsy have a greater risk of developing mental disorders?	100 (33.2)	103 (34.2)	98 (32.6)
4. Do you shake hands with a patient with epilepsy?	258 (85.7)	16 (5.3)	27 (9)
5. Do you allow your child to play with a child with epilepsy?	249 (82.7)	18 (6)	34 (11.3)
6. Do you allow your son to marry a girl with epilepsy?	194 (64.5)	45 (15)	62 (20.6)
7. Do you allow your daughter to marry a man with epilepsy?	188 (62.5)	46 (15.3)	67 (22.3)
8. Do you think that patients with epilepsy can have children?	263 (87.4)	8 (2.7)	30 (10)
9. Do you think that children of individuals with epilepsy have a greater chance of having malformations?	119 (39.5)	77 (25.6)	105 (34.9)
10. Do you think that patients with epilepsy are allowed to drive?	145 (48.3)	96 (33.2)	59 (19.7)
11. Do you think that patients with epilepsy can participate in sport activities?	226 (75.1)	31 (10.3)	44 (14.6)
12. Are you ready to work with a patient with epilepsy?	247 (82.1)	18 (6)	35 (11.6)
13. Do you think that a patient with epilepsy can get a high academic degree?	264 (87.7)	6 (2)	31 (10.3)
14. If you are an employer, do you allow a patient with epilepsy to be your employee?	246 (81.7)	17 (5.6)	38 (12.6)

Attitude score (14 items) score range 0–28, 0–16 (negative attitude), 17–28 (positive attitude).

**Table 5:** Factors associated with knowledge, awareness, and attitude scores.

Characteristics	Knowledge		*p*-value	Awareness		*P*-value	Attitude		*P*-value
	Poor (*n* = 38)	Good (*n* = 263)		Poor (*n* = 90)	Good (*n* = 211)		Negative (*n* = 36)	Positive (*n* = 263)	
Age									
18–30	26 (68.4%)	176 (66.9%)	0.980	64 (71.1%)	138 (65.4%)	0.422	29 (80.6%)	171 (65%)	0.165
31–40	8 (21.1%)	57 (21.7%)		19 (21.1%)	46 (21.8%)		4 (11.1%)	61 (23.2%)	
41–65	4 (10.5%)	30 (11.4%)		7 (7.8%)	27 (12.8%)		3 (8.3%)	31 (11.8%)	
Gender									
Male	13 (34.2%)	89 (33.8%)	0.964	26 (28.9%)	76 (36%)	0.232	12 (33.3%)	88 (33.5%)	0.998
Female	25 (65.8%)	174 (66.2%)		64 (71.1%)	135 (64%)		24 (66.7%)	175 (66.5%)	
Place of residence									
Mount Lebanon	6 (15.8%)	25 (9.5%)	0.778	15 (16.7%)	16 (7.6%)	0.011*	7 (19.4%)	24 (9.1%)	0.177
Bekaa	15 (39.5%)	136 (51.7%)		38 (42.2%)	113 (53.6%)		10 (6.7%)	139 (93.3%)	
Baalbek/Hermel	4 (10.5%)	18 (6.8%)		4 (4.4%)	18 (8.5%)		5 (22.7%)	17 (6.5%)	
North	2 (5.3%)	11 (4.2%)		5 (5.6%)	8 (3.8%)		2 (5.6%)	11 (4.2%)	
South	3 (7.9%)	17 (6.5%)		8 (8.9%)	12 (5.7%)		3 (8.3%)	17 (6.5%)	
Akkar	2 (5.3%)	12 (85.7%)		0 (0%)	14 (6.6%)		2 (5.6%)	12 (4.6%)	
Beirut	6 (15.8%)	38 (14.4%)		17 (18.9%)	27 (12.8%)		6 (16.7%)	38 (14.4%)	
Nabatieh	0 (0%)	6 (2.3%)		3 (3.3%)	3 (1.4%)		1 (2.8%)	5 (1.9%)	
Marital Status									
Single	22 (57.9%)	161 (61.2%)	0.329	58 (64.4%)	125 (59.2%)	0.699	22 (61.1%)	159 (60.5%)	0.793
Married	13 (34.2%)	94 (35.7%)		29 (32.2%)	78 (37%)		12 (33.3%)	95 (36.1%)	
Divorced	3 (7.9%)	8 (3%)		3 (3.3%)	8 (3.8%)		2 (5.6%)	9 (3.4%)	
Education									
University	21 (55.3%)	133 (50.6%)	0.093	46 (51.1%)	108 (51.2%)	0.417	21 (58.3%)	131 (49.8%)	0.023*
Post graduate	6 (15.8%)	89 (33.8%)		26 (28.9%)	69 (32.7%)		8 (22.2%)	87 (33.1%)	
Secondary	7 (18.4%)	31 (11.8%)		13 (14.4%)	25 (11.8%)		2 (5.6%)	36 (13.7%)	
Intermediate	2 (5.3%)	5 (1.9%)		1 (1.1%)	6 (2.8%)		2 (5.6%)	5 (1.9%)	
Primary	2 (5.3%)	5 (1.9%)		4 (4.4%)	3 (1.4%)		3 (8.3%)	4 (1.5%)	
Occupation									
Work in non-healthcare	17 (44.7%)	115 (43.7%)	0.015*	38 (42.2%)	94 (44.5%)	0.852	13 (36.1%)	117 (44.5%)	0.027*
Unemployed	13 (34.2%)	126 (47.9%)		44 (48.9%)	95 (45%)		16 (44.4%)	123 (46.8%)	
Retired	3 (7.9%)	3 (1.1%)		1 (1.1%)	5 (2.4%)		3 (8.3%)	3 (1.1%)	
Student in non-healthcare	5 (13.2%)	19 (7.2%)		7 (7.8%)	17 (8.1%)		4 (11.1%)	20 (7.6%)	
Current alcohol consumption									
No	36 (94.7%)	247 (93.9%)	0.842	86 (95.6%)	197 (93.4%)	0.463	33 (91.7%)	249 (94.7%)	0.464
Yes	2 (5.3%)	16 (6.1%)		4 (4.4%)	14 (6.6%)		3 (8.3%)	14 (5.3%)	
Current smoker									
No	24 (63.2%)	171 (65%)	0.822	62 (68.9%)	133 (63%)	0.330	23 (63.9%)	171 (65%)	0.894
Yes	14 (36.8%)	92 (35%)		28 (31.1%)	78 (37%)		13 (36.1%)	92 (35%)	
Monthly household income (Lebanese pounds)									
<750,000	5 (13.2%)	16 (6.1%)	0.079	10 (11.1%)	11 (5.2%)	0.123	5 (13.9%)	15 (5.7%)	0.212
750,000–2 million	17 (44.7%)	93 (35.4%)		33 (36.7%)	77 (36.5%)		14 (38.9%)	96 (36.5%)	
2–4 million	7 (18.4%)	99 (37.6%)		25 (27.8%)	81 (38.4%)		9 (25%)	97 (36.9%)	
>4 million	9 (23.7%)	55 (20.9%)		22 (24.4%)	42 (19.9%)		8 (22.2%)	55 (20.9%)	
Insurance type									
NSSF	10 (26.3%)	67 (25.5%)	0.065	27 (30%)	50 (23.7%)	0.567	14 (38.9%)	63 (24%)	0.249
Private	9 (23.7%)	68 (25.9%)		21 (23.3%)	56 (26.5%)		9 (25%)	67 (25.5%)	
COOP	4 (10.5%)	6 (2.3%)		4 (4.4%)	6 (2.8%)		1 (2.8%)	9 (3.4%)	
None	15 (39.5%)	122 (46.4%)		38 (42.2%)	99 (46.9%)		12 (33.3%)	124 (47.1%)	
Chronic illness									
No	31 (81.6%)	216 (82.1%)	0.934	79 (87.8%)	168 (79.6%)	0.091	31 (86.1%)	215 (81.7%)	0.520
Yes	7 (18.4%)	47 (17.9%)		11 (12.2%)	43 (20.4%)		5 (13.9%)	48 (18.3%)	
Attended lecture about epilepsy									
No	38 (100%)	191 (72.6%)	<0.001*	85 (94.4%)	144 (68.2%)	<0.001	34 (94.4%)	193 (73.4%)	0.006*
Yes	0 (0%)	72 (27.4%)		5 (5.6%)	67 (31.8%)		2 (5.6%)	70 (26.6%)	

^*^Statistically significant (*P* < 0.05). NSSF, national social security fund; COOP, cooperation healthcare fund.

**Table 6:** Logistic regression analysis.

*Logistic regression analysis taking the knowledge level as the dependent variable* (*R*^2^ = 0.182)			
	OR	95% CI	*P*-value
Education (Postgraduate vs. Bachelor*)	2.233	0.759–6.566	0.144
Occupation (employed vs. unemployed*)	0.619	0.224–1.715	0.357
Insurance (noninsured* vs. insured)	0.569	0.206–1.576	0.278
Attended lecture about epilepsy (yes vs. no*)	5.756	4.652–11.676	**<0.001**
*Logistic regression analysis taking the awareness level as the dependent* var*iable* (*R*^2^ = 0.158)			
Residency (Bekaa vs. others*)	1.534	0.909–2.589	0.109
Income (<2 million LBP* vs. greater)	1.263	0.747–2.134	0.384
Chronic illness (yes vs. no*)	1.986	0.946–4.169	0.07
Attended lecture about epilepsy (yes vs. no*)	4.936	3.062–10.566	**<0.001**
*Logistic regression analysis taking the attitude level as the dependent variable* (*R*^2^ = 0.161)			
Age (<30* vs. ≥30)	4.493	1.182–7.075	**0.027**
Residency (Bekaa vs. others*)	1.175	0.462–2.993	0.735
Education (Postgraduate vs. Bachelor*)	1.165	0.42–3.231	0.770
Occupation (employed vs. unemployed*)	1.185	0.424–3.306	0.746
Attended lecture about epilepsy (yes vs. no*)	5.187	1.687–9.761	**0.014**

*Reference group; OR, odds ratio; CI, confidence interval; numbers in bold indicate significant *p*-values.

## Discussion

The main objectives of the current study were to assess the Lebanese general population’s knowledge, awareness, and attitudes toward epilepsy and its associated factors given that it is a highly prevalent disease. In fact, a comprehensive review of epilepsy in the Middle East/North Africa (MENA) region in 2016 from 10 countries including Lebanon, estimated that median prevalence of epilepsy is 7.5/1000 but showed a lack of fundamental data on epilepsy prevalence or incidence (31). In addition, data from a recent report published by Bhalla et al. (31) accentuate the disease burden in the region; however, a lack of real numbers from Lebanon suggests a possible lower attention toward public health awareness and/or limited role of organizations in the country. This lack of attention may result in a substantial lack of knowledge and awareness as well as a negative disengagement with epileptic patients. This is particularly true as only one-quarter of the respondents attended lectures about epilepsy and greater than half agreed that their information on epilepsy is deficient.

### Epilepsy knowledge

The percentage of respondents who had a good knowledge of epilepsy (87.4%) was higher compared to Saudi individuals observed in Muthaffar and Jan (30) and Alhazzani et al. (32) studies. Conversely, a similar percentage of Brazilian residents (88%) were found to be knowledgeable about epilepsy (33). Some of the possible reasonings for this varied knowledge level may be the different knowledge items that were utilized in these studies, different methodology and study design, or the varied study population who may be differently exposed to awareness campaigns or public health interventions. A relatively good knowledge of the major etiologies of epilepsy: genetics (66.3%) and brain disease (59.8%) were noted in this study. However, the awareness of genetics as an etiological factor in India was only restricted to 10.4% of the respondents while infections (e.g., Neurocysticercosis) was only known to 3.3% of them (34). This may be in part due to the Arab’s increased familiarity with these attributes as etiological factors as highlighted by Bhalla et al. (31) to be the major causes of epilepsy in the Middle East. In the latter study, genetic origin (high consanguinity) was reported in 30 to 54% of epilepsy patients followed by brain disorders (e.g., Neurocysticercosis) in Eastern part of the MENA region (31). However, similar to the data from India (84.5%) and South Korea (82%), most of the respondents knew that epilepsy is not contagious (84.4%) (34, 35). However, a limited percentage of respondents were able to correctly describe epilepsy as a lifelong disease (61.5%), incurable (54.8%), and dangerous (47.8%). These results were consistent with those reported by Thacker et al. (34), where a significant proportion of Indian teachers (62%) believed that epilepsy was a curable disease (34). This highlights the pivotal need for awareness regarding these attributes as it shapes communities’ attitude toward the disease and expectation of the treatment efficacy. Of important note is that although a high degree of awareness was noted among the Lebanese population, a lot of misconceptions still exist regarding epilepsy. Furthermore, medications were thought to be the most effective treatment option (90.4%), while the role of surgery was thought to be limited (51.8%). In fact, according to the study conducted by Bhalla et al. (31), the surgical treatment of epilepsy is not very popular in the MENA region which resulted in some part in a decreased knowledge of this treatment option (31).

In addition, despite respondents’ awareness of clinical manifestations involved in a seizure: convulsion/shaking (75.4%), sudden loss of consciousness (67.1%), and behavioral change (53.5%), this study revealed that participants were not aware of the appropriate response when dealing with someone having seizure as only 60.5% were aware of the need to remove nearby sharp objects, a lower percentage (58.5%) recognized that they should call an ambulance or protect the patient’s head (55.8%). Similarly, taking patients out of danger was a priority for 92.8% of the respondents in Alsohibani et al. (28) study. Nevertheless, respondents from other studies would go for forcing medications down a patient’s throat or using herbal medicine or seeking a spiritual healer (32).

### Awareness toward epilepsy

Despite the suggested facts, most of the respondents had good awareness (70.1%) of epilepsy. In this regard, almost half of them (48.2%) had previously witnessed epilepsy which mirrors the percentages reported previously by Alsohibani et al. (28) and Muthafar et al. (30) as 48.4 and 50%, respectively, suggesting an approximately similar prevalence in both Lebanon and Saudi Arabia. Moreover, around one-third (37.9%) had a relative with epilepsy, and around one-quarter (24.6%) had a close friend with epilepsy. Those findings are higher than those reported in Iran where only 23.9% had a relative with epilepsy although a higher proportion was noted among those who knew someone with the disease (58.1%) (36). In fact, being in contact with someone with epilepsy can be a great source of awareness to individuals themselves, in addition to the efforts invested by public media and healthcare personnel as noted in a study conducted by Thacker et al. (34).

### Attitudes toward epilepsy

In addition, it was noted that respondents had good attitudes toward epileptic patients (88%), where the majority of them (83.4%) felt that epileptic patients are as intelligent as other people, 87.2% thought they can earn a high academic degree, 75.1% believed that they can participate in sport activities, and 87.4% said that they can have children. In addition, 85.7% of the respondents had no problem shaking hands of epileptic patients, working with them (82.1%), and employing them (81.7%). Similarly, the majority of the respondents in Alhazzani et al. (32) study were willing to work with epileptic patients (80.9%). However, around two-thirds of the respondents (67.8%) believed that epileptic patients are mentally ill and around half (48.3%) agreed that they should not drive.

In line with the findings of our study, the majority of the respondents in other studies conducted in the Middle East agreed that epileptic patients should not be excluded from work environments and had a good attitude toward children affected by the disease (28, 32, 36–38). Nevertheless, people from India still have doubts about the intelligence level of epileptic patients where approximately one-third of the respondents considered epileptic patients have a below-average intelligence (34). While, only a limited percentage (6%) of respondents in this study refused to have their children get involved with others having the disease which is even lower than that reported by Alsohibani et al. (28) (7.9%). Also, more respondents in this study (62.5%) were open to their son’s and daughter’s marriage to someone with epilepsy compared to data from Saudi Arabia and India (28, 34).

Finally, almost half of the participants felt the desire to help seizing patients (55.8%) while the rest were primarily frightened. Similarly, half of the teachers from Thailand were reluctant to deliver first aid management (39). More importantly, the majority of those who offered first aid in Thailand (86.4%) did it improperly (e.g., inserted a spoon, held a child down, or put a child on his/her back) (39). Similarly, a substantial proportion of participants in this study opt to hold a patient’s tongue (36.9%) or to restrict a patient’s movement (41.9%).

### Limitations

Although the included sample size was large and diverse enough, it may not be sufficient to draw definitive conclusions or ensure the generalizability of the findings to the broader Lebanese population. However, the current study still serves as a preliminary investigation, highlighting important trends and areas for further exploration and future research could benefit from larger, more representative samples to enhance the generalizability of the results. Additionally, the snowball sampling technique could have introduced selection bias, as participants were likely recruited from similar social networks, potentially limiting sample diversity. The reliance on self-reported data is another limitation, as it is subject to recall bias and social desirability bias, which could impact the accuracy of the responses. Moreover, the online recruitment method may have excluded individuals without internet access or those less familiar with digital platforms, restricting the sample to a more digitally literate group. Although the questionnaire was offered in both English and Arabic, some nuances may have been lost in translation, potentially affecting participants’ understanding of the questions. Lastly, as this is a cross-sectional study, it captures knowledge, awareness, and attitudes at a single point in time, limiting the ability to establish causal relationships.

## Conclusion

Overall, adequate knowledge and awareness toward epilepsy have been detected in this study and a positive attitude has been noted toward epileptic patients in Lebanon. However, major misconceptions have been observed which warrants a strong public education toward a proper first aid seizure management which will further improve the public’s attitude toward the disease. This will enhance the confidence of the general public in approaching seizing patients and providing the adequate care which can result in improved healthcare in return.

## Data Availability

The raw data supporting the conclusions of this article will be made available by the authors, without undue reservation.
